# Surgical and survival outcomes of hemispheric low-grade glioma in Ethiopia: a multicenter retrospective cohort study

**DOI:** 10.3389/fonc.2026.1899634

**Published:** 2026-10-28

**Authors:** Moti Belay Daba, Yemisirach Bizuneh Akililu

**Affiliations:** 1 Marshall University Joan C. Edwards School of Medicine, Huntington, WV, United States; 2 Department of Neurosurgery, Tikur Anbessa Specialized Hospital, Addis Ababa University, Addis Ababa, Ethiopia

**Keywords:** Ethiopia, extent of resection, hemispheric glioma, low-grade glioma, overall survival, seizure outcome

## Abstract

**Background:**

Outcome data for hemispheric low-grade glioma from resource-limited settings remain limited. This study describes surgical, seizure, and survival outcomes among patients treated at two tertiary centers in Ethiopia.

**Objective:**

To describe surgical, seizure, and survival outcomes and explore factors associated with overall survival among patients undergoing surgery for hemispheric low-grade glioma.

**Methods:**

We conducted a multicenter retrospective cohort study of patients who underwent surgery for hemispheric low-grade glioma at two tertiary hospitals in Addis Ababa, Ethiopia, between January 2015 and December 2020. Demographic, clinical, radiologic, operative, histopathologic, treatment, and follow-up data were analyzed. Overall survival was estimated using the Kaplan-Meier method. Associations with survival were explored using univariable Cox proportional hazards analyses. Given the small number of events, survival-factor analyses were considered exploratory.

**Results:**

Thirty-four patients constituted the clinical cohort, with a median age of 39 years. Gross-total resection was performed in 23 (67.6%) patients. Thirty-one patients had sufficient information for time-to-event analysis, among whom 13 deaths were observed. Kaplan-Meier estimated overall survival was 96.7% at 1 year, 67.2% at 2 years, and 53.5% at 3 years. No patients remained under observation at 60 months; therefore, a 5-year survival estimate was not reported. In exploratory univariable analyses, age>40 years, contrast enhancement, and subtotal resection were associated with higher mortality, whereas recorded postoperative chemoradiation was associated with lower mortality. Estimates were imprecise, with wide confidence intervals. Among 23 patients with documented postoperative seizure outcomes, 16 (69.6%) achieved Engel class I status.

**Conclusion:**

This multicenter cohort provides real-world surgical and survival data for hemispheric low-grade glioma care in Ethiopia. Several clinical and treatment characteristics were associated with survival, but the small number of events, incomplete follow-up, missing data, and absence of molecular classification preclude definitive conclusions regarding independent prognostic effects. Larger prospective studies incorporating contemporary molecular diagnostics and standardized longitudinal follow-up are needed.

## 1 Introduction

Low-grade gliomas are infiltrative primary central nervous system tumors characterized by a relatively prolonged clinical course but substantial long-term neurologic morbidity and mortality. Although they grow more slowly than high-grade gliomas, diffuse low-grade gliomas remain biologically progressive lesions capable of malignant transformation and neurologic deterioration (1–3).

Surgical resection remains a central component of management for hemispheric low-grade glioma. Maximal safe resection provides tissue for diagnosis, reduces tumor burden, may improve seizure control, and has been associated with improved outcomes in observational studies (2, 4–7). Tumors involving eloquent cortical or subcortical regions, however, pose important operative challenges and may limit the achievable extent of resection.

Management in high-resource settings increasingly incorporates molecular diagnostics, advanced neuroimaging, neuronavigation, intraoperative neurophysiologic monitoring, and awake mapping. Access to these technologies and to longitudinal multidisciplinary oncologic care may be inconsistent in resource-limited settings (2, 4, 5, 8). Published neuro-oncology outcome data from sub-Saharan Africa remain limited, and available regional reports have often focused on broad intracranial tumor patterns rather than longitudinal low-grade glioma outcomes (9, 10).

This study therefore aimed to describe the clinical characteristics, surgical treatment, seizure outcomes, and overall survival of patients undergoing surgery for hemispheric low-grade glioma at two tertiary centers in Addis Ababa, Ethiopia, and to explore clinical and treatment factors associated with survival.

## 2 Methods

### 2.1 Study design and setting

This multicenter retrospective cohort study was conducted at Tikur Anbessa Specialized Hospital and Myung Sung Christian Medical Center in Addis Ababa, Ethiopia. Patients undergoing surgical treatment for hemispheric low-grade glioma between January 1, 2015 and December 31, 2020 were considered for inclusion.

### 2.2 Study population and patient flow

Patients were eligible if they had a histopathologic diagnosis of hemispheric low-grade glioma and available clinical documentation. WHO grade I tumors and alternative tumor diagnoses were excluded. Seventy-five potentially eligible records were initially identified. The recovered patient-level analytic dataset contained 34 patients. Detailed patient-level information permitting reliable reconstruction of the specific reasons for non-inclusion of the remaining 41 records was not preserved; these reasons were therefore not retrospectively assigned.

For the primary survival analysis, patients required sufficient information to define an event time or censoring time. Of the 34 patients in the recovered clinical cohort, 31 met this requirement. Two patients recorded as alive lacked a follow-up duration, and one patient recorded as deceased lacked a time-to-death. These three patients were excluded from the primary time-to-event analysis rather than assigning an assumed survival time (**Figure 1**).

**Figure 1:**
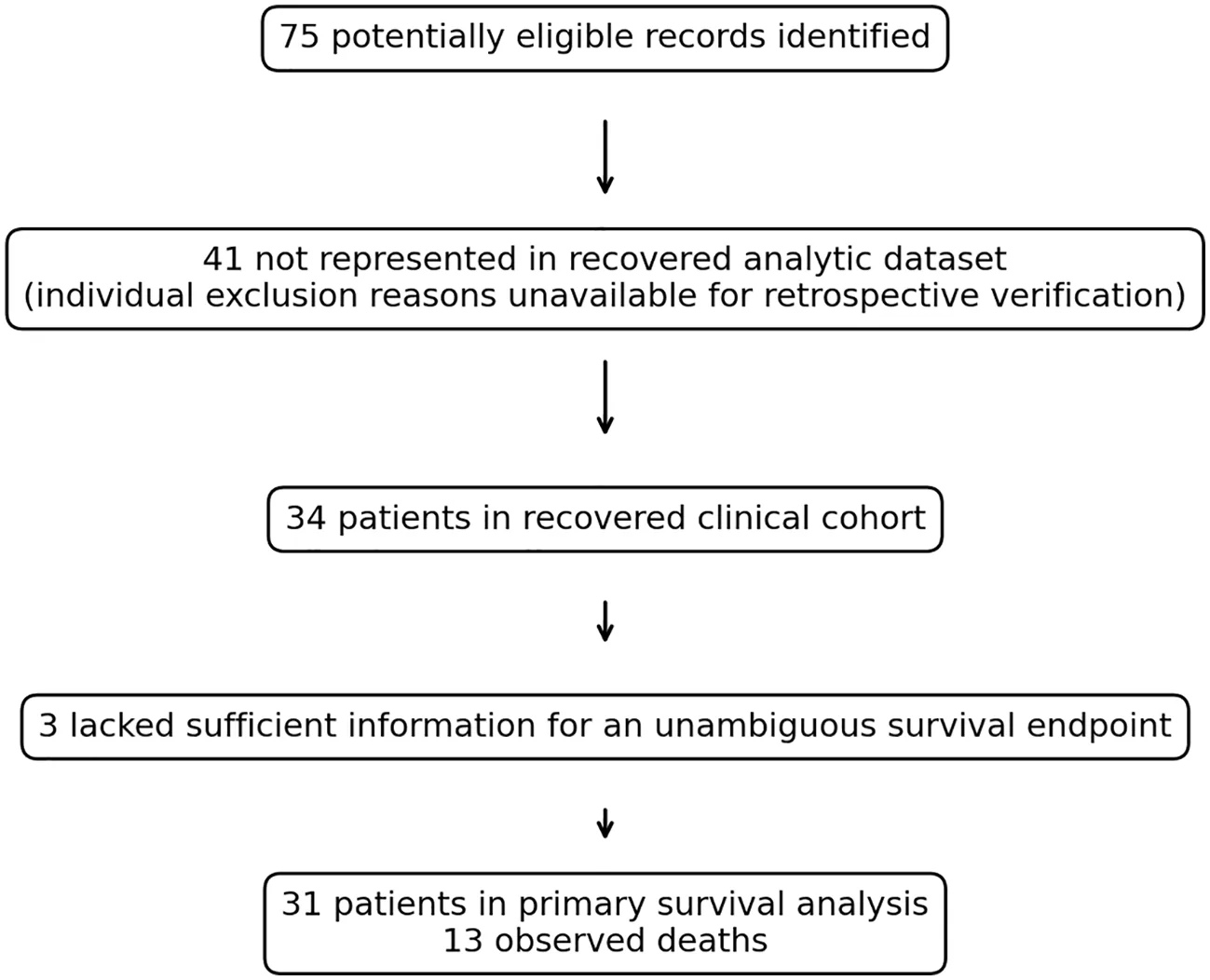
Verified patient flow from potentially eligible records to the primary survival cohort. The recovered analytic dataset did not preserve sufficient patient-level information to retrospectively assign specific exclusion reasons to the 41 records not represented in the analytic cohort.

### 2.3 Data collection and variable definitions

The recovered analytic dataset contained demographic, clinical, radiologic, operative, histopathologic, treatment, seizure, and follow-up variables. Variables used in the revised analysis included age, sex, Karnofsky Performance Status (KPS), tumor diameter, contrast enhancement, histopathologic diagnosis, extent of resection, postoperative chemoradiation status, seizure outcome, follow-up duration, vital status, and time to death.

Age was categorized as <=40 or >40 years, KPS as <80 or >=80, and tumor diameter as <6 or >=6 cm. Extent of resection was categorized as gross-total resection (GTR) or subtotal resection (STR).

Histopathologic diagnoses reflect the morphology-based classification used during the study period. Molecular characterization, including IDH mutation and 1p/19q codeletion status, was not routinely available.

Postoperative treatment was recorded in the source dataset as chemoradiation status. Detailed information regarding individual chemotherapy agents, radiotherapy dose or fractionation, treatment sequencing, and treatment completion was not consistently preserved and was not retrospectively inferred.

### 2.4 Outcomes

The primary outcome was overall survival, defined as the interval from the first tumor operation to death or last documented follow-up. For patients with documented death and an available time-to-death, the recorded interval from surgery to death was used as the event time. Patients recorded as alive were censored at their last documented follow-up. Postoperative seizure outcome was summarized using the recorded Engel classification at last documented follow-up.

### 2.5 Statistical analysis

Continuous and categorical variables were summarized descriptively. Denominators are reported when observations were missing. Overall survival was estimated using the Kaplan-Meier method, with numbers at risk reported at prespecified time points. Univariable Cox proportional hazards models were used to explore associations between selected clinical and treatment characteristics and overall survival. Hazard ratios (HRs), 95% confidence intervals (CIs), and p-values are reported. Because only 13 deaths occurred in the primary survival cohort and several predictors contained sparse or missing data, the previously specified multivariable Cox model was not retained because of the high risk of overfitting and unstable coefficient estimates. Survival-factor analyses were therefore considered exploratory. KPS was not assigned a conventional Cox HR because no deaths occurred in the KPS >=80 group among patients with available KPS and survival data, resulting in separation. Statistical analyses were reconstructed from the recovered patient-level dataset.

### 2.6 Ethical considerations

The study was approved by the Institutional Review Board of Addis Ababa University, College of Health Sciences, Department of Surgery, and was conducted in accordance with applicable local legislation and institutional requirements. Written informed consent for participation was not required in accordance with the approved retrospective study procedures. No potentially identifiable images or patient-level data are presented in this manuscript.

## 3 Results

### 3.1 Patient characteristics

Thirty-four patients constituted the recovered clinical cohort. Median age was 39 years; 19 patients (55.9%) were aged <=40 years and 15 (44.1%) were aged >40 years. Sixteen patients (47.1%) were male and 18 (52.9%) were female. KPS was available for 30 patients: 14/30 (46.7%) had KPS <80 and 16/30 (53.3%) had KPS >=80 (**Table 1**).

**Table 1:** Baseline demographic and functional characteristics.

Characteristic	n (%)
Total cohort	34
Median age	39 years
Age <=40 years	19 (55.9)
Age >40 years	15 (44.1)
Male	16 (47.1)
Female	18 (52.9)
KPS <80	14/30 (46.7)
KPS >=80	16/30 (53.3)
KPS missing	4

### 3.2 Radiologic characteristics

Tumor diameter was <6 cm in 20/34 patients (58.8%) and >=6 cm in 14/34 (41.2%). Contrast-enhancement status was available for 30 patients; enhancement was present in 11/30 (36.7%) and absent in 19/30 (63.3%). Enhancement status was missing for four patients (**Table 2**).

**Table 2:** Radiologic characteristics.

Radiologic characteristic	n (%)
Tumor diameter <6 cm	20 (58.8)
Tumor diameter >=6 cm	14 (41.2)
Contrast enhancement present	11/30 (36.7)
Contrast enhancement absent	19/30 (63.3)
Enhancement status missing	4

### 3.3 Surgical treatment and postoperative therapy

GTR was recorded in 23/34 patients (67.6%) and STR in 11/34 (32.4%). Postoperative chemoradiation status was available for 31 patients; 25/31 (80.6%) had postoperative chemoradiation recorded and 6/31 (19.4%) did not. Treatment status was missing for three patients (**Table 3**). Detailed regimen-level information was not available in the recovered dataset.

**Table 3:** Surgical and postoperative treatment characteristics.

Treatment characteristic	n (%)
Gross-total resection	23 (67.6)
Subtotal resection	11 (32.4)
Postoperative chemoradiation recorded	25/31 (80.6)
No postoperative chemoradiation	6/31 (19.4)
Chemoradiation status missing	3

### 3.4 Histopathology

Historical morphology-based diagnoses consisted of diffuse fibrillary astrocytoma in 13/34 patients (38.2%), diffuse gemistocytic astrocytoma in 8/34 (23.5%), and oligodendroglioma in 13/34 (38.2%) (**Table 4**). Molecular information required for contemporary integrated WHO classification was unavailable.

**Table 4:** Historical morphology-based histopathologic classification.

Historical morphology-based diagnosis	n (%)
Diffuse fibrillary astrocytoma	13 (38.2)
Diffuse gemistocytic astrocytoma	8 (23.5)
Oligodendroglioma	13 (38.2)

### 3.5 Overall survival

Of the 34 patients in the clinical cohort, 31 had sufficient information to establish survival time and censoring status and were included in the primary survival analysis. Thirteen deaths were observed. Kaplan-Meier estimated overall survival was 96.7% at 1 year, 67.2% at 2 years, and 53.5% at 3 years. The numbers at risk at 12, 24, and 36 months were 28, 15, and 5, respectively. Only one patient remained at risk at 48 months, and no patients remained under observation at 60 months; therefore, a 5-year survival estimate was not reported (**Figure 2**).

**Figure 2:**
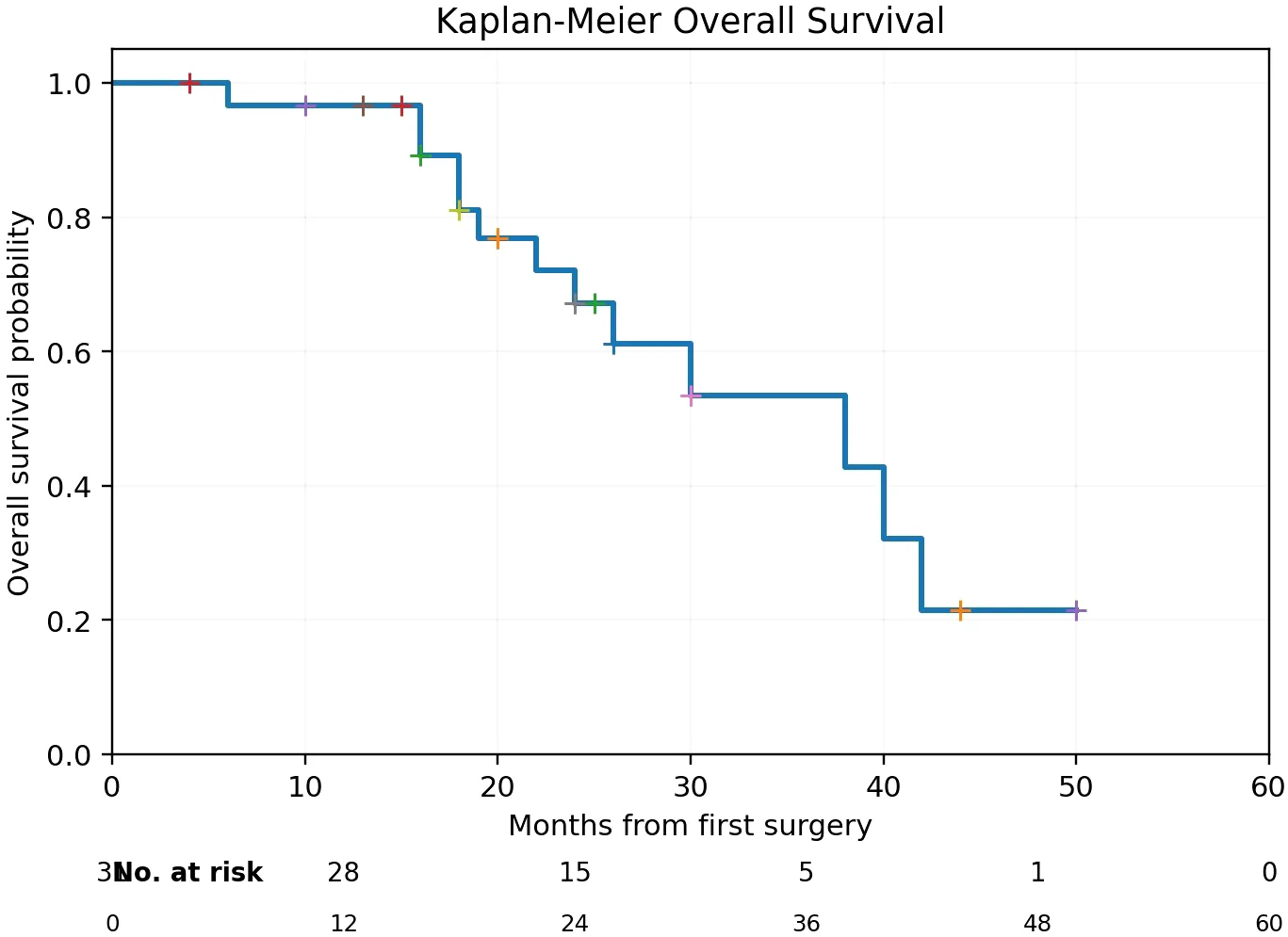
Kaplan-Meier overall survival curve for the primary survival cohort (n=31; 13 deaths). Censoring is indicated by plus signs. Numbers at risk are shown below the curve.

### 3.6 Exploratory factors associated with overall survival

Given the limited number of observed deaths, prognostic analyses were considered exploratory. In univariable Cox analyses, age >40 years was associated with higher mortality (HR 3.89, 95% CI 1.18-12.77; p=0.025). Contrast enhancement was associated with higher mortality (HR 10.80, 95% CI 1.33-87.98; p=0.026), although the wide confidence interval reflected substantial uncertainty. STR compared with GTR was associated with higher mortality (HR 4.41, 95% CI 1.33-14.56; p=0.015). Recorded postoperative chemoradiation was associated with lower mortality (HR 0.21, 95% CI 0.05-0.84; p=0.027). Tumor diameter >=6 cm (HR 2.31, 95% CI 0.71-7.56; p=0.166) and fibrillary astrocytoma compared with other recorded histologies (HR 1.78, 95% CI 0.54-5.94; p=0.346) were not statistically significant.

Among the 27 patients with available KPS and survival data, all nine observed deaths occurred among patients with KPS <80 and none among those with KPS >=80. This separation precluded a stable conventional Cox estimate; KPS is therefore described without a hazard ratio (**Table 5**).

**Table 5:** Exploratory univariable Cox proportional hazards analyses of overall survival.

Variable	N	Deaths	HR (95% CI)	p-value
Age >40 vs <=40 years	31	13	3.89 (1.18-12.77)	0.025
Tumor diameter >=6 vs <6 cm	31	13	2.31 (0.71-7.56)	0.166
Contrast enhancement present vs absent	27	9	10.80 (1.33-87.98)	0.026
Subtotal vs gross-total resection	31	13	4.41 (1.33-14.56)	0.015
Postoperative chemoradiation: yes vs no	30	12	0.21 (0.05-0.84)	0.027
Fibrillary astrocytoma vs other recorded histologies	31	13	1.78 (0.54-5.94)	0.346
KPS <80 vs >=80	27	9	Not estimable*	–

*No deaths occurred in the KPS >=80 group among patients with available KPS and survival data, resulting in separation and an unstable conventional Cox estimate.

### 3.7 Seizure outcomes

Preoperative seizure status was available for 33 patients, of whom 25/33 (75.8%) had seizures. Postoperative Engel outcome was documented for 23 patients. Sixteen of 23 (69.6%) achieved Engel class I status, while 7/23 (30.4%) had Engel class II-IV outcomes at last documented follow-up (**Table 6**).

**Table 6:** Seizure presentation and documented postoperative seizure outcome.

Seizure variable	n (%)
Preoperative seizure	25/33 (75.8)
No preoperative seizure	8/33 (24.2)
Preoperative seizure status missing	1
Engel outcome documented	23
Engel class I	16/23 (69.6)
Engel class II-IV	7/23 (30.4)

### 3.8 Postoperative morbidity data

Postoperative complication variables in the recovered dataset contained substantial missing information, precluding reliable estimation of an overall complication incidence. These outcomes were therefore not used for inferential analysis and an aggregate complication rate was not reported.

## 4 Discussion

This multicenter retrospective cohort describes surgical, seizure, and survival outcomes among patients treated for hemispheric low-grade glioma at two tertiary centers in Addis Ababa, Ethiopia. Following re-evaluation of the recovered patient-level dataset, 34 patients constituted the clinical cohort and 31 had sufficient information for time-to-event analysis. Thirteen deaths were observed. Kaplan-Meier estimated overall survival was 96.7% at 1 year, 67.2% at 2 years, and 53.5% at 3 years. Because the risk set became very small during longer follow-up and no patients remained under observation at 60 months, a 5-year survival estimate was not reported.

These data provide a description of low-grade glioma care in a setting where access to contemporary neuro-oncology resources has historically been constrained. Current management increasingly incorporates molecular classification, advanced imaging, intraoperative mapping, neurophysiologic monitoring, and multidisciplinary oncologic care (2, 4, 5, 8). Several of these resources were unavailable or inconsistently accessible during the study period. The present findings should therefore be interpreted primarily as real-world descriptive and exploratory data from that resource context rather than as a definitive prognostic model.

GTR was recorded in 23 of 34 patients (67.6%). In exploratory univariable analysis, STR was associated with higher mortality compared with GTR. This direction is consistent with observational literature supporting maximal safe resection in diffuse low-grade glioma (4–7). However, extent of resection was not randomized and may reflect tumor location, eloquence, infiltrative characteristics, patient functional status, and other factors that may themselves influence survival. The present study therefore cannot establish a causal survival benefit from GTR.

Contrast enhancement was associated with mortality in exploratory analysis. Enhancement can be associated with more aggressive biological behavior, but the estimate in this cohort was imprecise, with a very wide confidence interval. Older age was also associated with poorer overall survival, consistent with historical prognostic literature (11–15). KPS showed a marked pattern: no deaths occurred among patients with KPS >=80 in the analyzable subset. Because of the small sample, missing KPS values, and complete separation of events across KPS categories, the magnitude of this association could not be estimated reliably.

Recorded postoperative chemoradiation was associated with lower mortality in exploratory univariable analysis. This observation requires cautious interpretation. Treatment allocation was nonrandomized and may have been influenced by age, functional status, extent of resection, tumor characteristics, treatment access, and other clinical factors. In addition, the recovered dataset did not contain sufficiently detailed information regarding chemotherapy agents, radiotherapy dose or fractionation, sequencing, or treatment completion. The observed association should therefore not be interpreted as a comparative estimate of efficacy for a specific postoperative regimen (2, 8, 16).

Tumor diameter >=6 cm and fibrillary astrocytoma histology were not statistically significant in the reconstructed univariable analyses. These findings should not be interpreted as evidence that tumor size or tumor biology lacks prognostic importance; rather, this small cohort does not provide sufficiently precise evidence to establish those associations.

### 4.1 Histopathology in the context of contemporary classification

Histopathologic diagnoses in this cohort reflect morphology-based classification used during the study period. The recovered dataset contained diagnoses of diffuse fibrillary astrocytoma, diffuse gemistocytic astrocytoma, and oligodendroglioma. Routine molecular characterization, including IDH mutation and 1p/19q codeletion status, was unavailable. Consequently, these historical diagnoses cannot be directly mapped onto contemporary integrated adult-type diffuse glioma entities and should be interpreted within their historical diagnostic context.

### 4.2 Seizure outcomes

Among patients with documented postoperative Engel outcomes, 69.6% achieved Engel class I status. Surgical treatment of low-grade glioma can provide meaningful seizure control, and prior studies have identified tumor and treatment factors associated with postoperative seizure outcomes (17). However, postoperative Engel outcome was available for only 23 patients in this cohort, limiting more detailed inference.

### 4.3 Limitations

This study has several important limitations. First, its retrospective design and small sample size limit statistical precision and increase susceptibility to selection bias and residual confounding. Of 75 potentially eligible records initially identified, 34 constituted the recovered analytic cohort. Detailed patient-level information permitting reconstruction of the reasons for non-inclusion of all 41 records was not preserved, so selection and attrition bias cannot be excluded.

Second, follow-up was incomplete. Of the 34 patients, 31 had sufficient information to establish an unambiguous survival endpoint. The number at risk declined substantially beyond 3 years, precluding a robust estimate of longer-term survival. Third, only 13 deaths with documented survival times were available for survival modeling. This event count was insufficient to support the previously specified multivariable Cox model without substantial risk of overfitting; accordingly, the revised survival-factor analyses are exploratory univariable associations rather than independently validated prognostic effects.

Fourth, several variables contained missing observations, including KPS, contrast enhancement, treatment status, and postoperative outcomes. Postoperative complication variables were particularly incomplete, preventing reliable estimation of an overall complication incidence. Fifth, molecular testing including IDH mutation and 1p/19q codeletion status was not routinely available, and histopathologic categories therefore reflect historical morphology-based diagnoses. Sixth, extent of resection was based on the clinical and radiologic assessment available during the study period rather than standardized volumetric analysis.

Finally, detailed postoperative chemotherapy and radiotherapy regimen information was not consistently preserved, and treatment associations remain susceptible to selection bias and residual confounding.

## 5 Conclusion

This multicenter retrospective cohort provides descriptive surgical, seizure, and survival data for hemispheric low-grade glioma treated at two tertiary centers in Ethiopia. Among patients with sufficient follow-up information, estimated overall survival was 67.2% at 2 years and 53.5% at 3 years; longer-term survival could not be reliably characterized because of the small number of patients remaining under observation. Exploratory analyses identified associations between survival and age, contrast enhancement, extent of resection, and recorded postoperative chemoradiation, while KPS showed marked event separation. The small number of events, incomplete follow-up, missing data, and absence of contemporary molecular classification preclude definitive conclusions regarding independent prognostic effects. Larger prospective studies incorporating standardized follow-up, molecular diagnostics, and detailed treatment information are warranted.

## Data Availability

The original contributions presented in the study are included in the article/supplementary material. Further inquiries can be directed to the corresponding author.
